# Supine tubeless upper pole PCNL under spinal anaesthesia: Safety, feasibility and outcomes from a tertiary endourology centre

**DOI:** 10.1080/20905998.2024.2309780

**Published:** 2024-01-26

**Authors:** Nitesh Kumar, Bhaskar Somani

**Affiliations:** a Consultant Urological Surgeon, Ford Hospital and Research Centre, Patna, India; b Urology, University Hospital Southampton NHS Foundation Trust, Southampton, UK

**Keywords:** PCNL, supine, spinal, ureteroscopy, kidney calculi

## Abstract

**Objective:**

Supine Percutaneous Nephrolithotomy (PCNL) is being taken up by many urologists in recent times, but there is a tendency to shift to prone PCNL for upper pole puncture. We analyzed the safety, feasibility and outcomes of upper pole access in Supine Percutaneous Nephrolithotomy (sPCNL).

**Materials and methods:**

A retrospective review of all patients undergoing sPCNL at a tertiary care center was done from January 2021 to December 2022. Data collection was done from the maintained imaging, laboratory and hospital records. All cases with complete data on upper pole access were included. Data analysis was done with Xlstat2021.

**Results:**

50 patients with upper pole access were included (64%, 32 with single access and 36%, 18 with multiple accesses). The mean stone size was 23.88 ± 9.99, mean HU was 1093 ± 232.83, and the mean operative duration was 67.92 ± 34.62. Stone clearance rate was 98.82%, with all procedures performed tubeless.

The mean haemoglobin drop was 0.75 ± 0.42 gm/dl with 2 (4%) patients needing a blood transfusion. The overall complication rate was 22% with only 1 Clavien Dindo III complication (1 pleural injury and hydrothorax needing USS guided aspiration) and others being Clavien Dindo I/II complications.

**Conclusion:**

Supine PCNL is a feasible and safe approach for upper pole access. While the procedure can be done tubeless, these procedures must be done in experienced endourology units.

## Introduction

Percutaneous Nephrolithotomy (PCNL) is the standard minimal invasive treatment for large and complex renal stones [1]. It has replaced open surgery after its first description in 1976 by Fernstrom and Johansson [2].

Prone (pPCNL) position is preferred by most of the urologist worldwide for performing PCNL. It provides direct access to the pelvicalyceal system (PCS) through a puncture in the brodel’s line, larger area for puncture, good stone clearance, lower risk of injury to abdominal viscera, and familiarity due to training [3].

In 1987, Valdivia Uria described his simplified PCNL technique in supine position (sPCNL). He described several advantages of this position over prone technique. There was low impact on circulation and accessibility to airway, making it a safer from the anesthesia perspective too [4].

sPCNL was very helpful in pediatric, geriatric, obese/overweight, spinal deformity patients and those who are debilitated. There was low intrarenal pressure, rapid removal of stones by gravity, more comfortable patient and surgeon positioning during surgery and less radiation to the surgeon, making it faster and safer [5].

However, hyper mobility of the kidney, collapsed pelvicalyceal system (PCS) during surgery, small available area for puncture, and difficult upper pole access were the disadvantages, and it did not attract many urologists at the outset [6]. Gradually with advent of Retrograde Intrarenal Surgery (RIRS) and Endoscopic Combined Retrograde Intrarenal Surgery (ECRIS), the adoption rates of supine PCNL have increased worldwide [7].

Most of the technical difficulties of the procedure were overcome with experience. Many urologists revert back to pPCNL in complex scenarios especially the cases which requires upper pole access. We retrospectively analysed our data from large case series, for all cases requiring upper pole puncture, for evaluating the feasibility and safety of upper pole access in sPCNL. This is one of the first papers on upper pole puncture in sPCNL performed as a tubeless procedure.

## Material and methods

A retrospective analysis of all patients undergoing sPCNL at Ford Hospital and research centre was done from January 2021 to December 2022. Ethical approval was obtained (FHRC/IEC/AUG-2023/001). The inclusion criteria were all patients with upper pole puncture done as a sole tract or as a part of multiple tracts. The exclusion criteria were patients with incomplete data or follow-up. A CT scan was performed for all patients pre-operatively.

Baseline demographic data along with the following parameters were recorded: age, sex, body mass index (BMI), comorbidities, side, size, number of stones, stone location, Hounsfield Unit (HU), renal anomalies, previous surgery, type of anesthesia, number, size and location of tracts, lithotripsy modality, duration of surgery, exit strategy, stone clearance, irrigation fluid used, hemoglobin (Hb) drop, transfusion rate, hospital stay, and complications. Data analysis was done with XlStat2021 software.

Spinal anesthesia (SA) was administered for all patients. A 25 Gauge spinal needle was used, and 3 ml of Bupivacaine (Anawin heavy) was instilled; 1 ml (50 microgram) of fentanyl was used as an adjuvant in cases with larger stone sizes.

**Figure 1.**
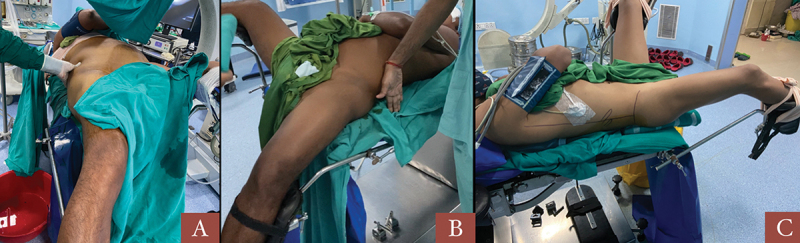
Patient position and marking of landmarks; A- view from leg end, B- Contralateral leg flexed at knee, C- Ipsilateral leg extended.

After achieving the necessary anesthesia effect, patients were positioned in a modified supine position. The contralateral leg was kept in lithotomy position and ipsilateral leg was kept either straight or flexed at knee, the contralateral arm was kept on an armrest with abduction less than 90 degrees and patient was asked to hold the contralateral shoulder with the ipsilateral arm. Patient was brought to the edge of the table and two small bolsters were kept, one below the scapula and the other below the buttocks (Figure 1). Tilt of trunk was kept to minimum to avoid overlap of pelvicalyceal system (PCS) and stones with the spine position.

The operation theatre and instruments were setup in a very particular way to help surgeon do the procedure with minimal assistance. The camera trolley was on the patient’s head end, c-arm machine in center and the screen of c-arm on foot end (all three on the contralateral side of the stone). The c-arm foot switch was kept on floor (head end), the laser and lithotripsy/laser foot pedal on foot end of the patient. Assistance from operating theatre (OT) floor staff was limited to changing saline and water pressure regulation.

**Figure 2.**
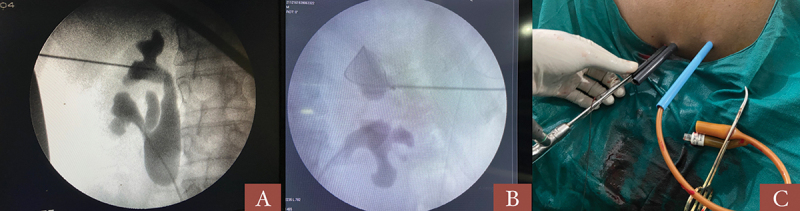
Different scenarios showing upper pole puncture; A- stone in upper pole infundibulum, B- stone in upper pole in bifid PCS, C- external view in multitract puncture.

**Figure 3.**
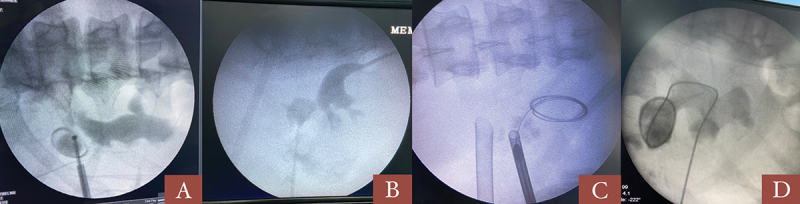
Different scenarios showing upper pole puncture; A- stone in upper pole diverticulum, B- isolated stone in upper pole, C- additional upper pole puncture for multiple secondary stones, D- upper pole access in staghorn stone.

A 5F ureteric catheter was placed, and retrograde pyelogram (RGP) was performed. The lateral upper calyx was punctured in all cases with supra-eleventh rib puncture in some cases. Deep inspiration and breath holding was not done during the puncture. Supine monoplanar technique (c-arm in 0 degrees) was used in all cases and biplanar was used only after three failed initial attempts. Mini (16.5/17.5 F, 20F, 22F) or standard 24 F tract/amplatz sheath was used depending on the stone size (Figures 2 and 3).

Holmium 60W Laser (Cyber Ho Quanta) with initial settings of 1.5J, 10 Hz lithotripsy mode (increased to maximum of 2J and 10 Hz) was used for breaking the stone. Pneumatic lithotripter was used in large volume stones and bigger tracts (Nidhilith digi) 2.5 mm probe for standard PCNL and 1 mm probe for mini (occasionally) with settings of 1.5 kg/cm2 pressure and a frequency of 4. The fragments were removed mostly by gravity and forceps when required. After clearing the stone, a 5F, 26 centimeter (cm) Double J (DJ) stent was placed and cystoscopy was done to confirm the bladder end coil of the DJ stent. Tubeless exit was done in all cases and a single staple applied to the puncture site. Intraoperative parameters were recorded.

Postoperative parameters and complications were recorded. Xray abdomen was done on the next day morning to check on the residual stones. Patients were discharged on the first or second post-operative day after removing the stapler and foley catheter, depending on their clinical condition. All patients were reviewed after a month with Ultrasonography (USG) of abdomen to document and assess stone clearance and the DJ stent was removed. Patients with residual stones were counselled for retrograde intrarenal surgery (RIRS) or a re-look PCNL. A repeat follow up was done at 3 months with USG abdomen. Stone-free rate was defined as complete clearance of stone endoscopically or presence of fragments <2 mm on the follow-up imaging. Complications were graded according to the Clavien-Dindo classification system.

## Results

In our retrospective analysis, upper pole access was made in 53 patients. Two patients were excluded as they were lost to follow up and one was excluded due to incomplete data entry.

The patient demographics and stone characteristics are presented in Table 1. The mean age was 39.82 ± 14.99 (range:14–69 years), male to female ratio was 1.38:1, and the mean BMI was 24.2 ± 3.47 (range:18–32). About 44% of the patients have one or more associated co-morbidities and 60% of stones were on the right side.

**Table 1.** Patient demographics and stone characteristics.

Parameter	Results (*n* = 50)	Only upper pole access (*n* = 32)
Mean age ± SD (range)	39.82 ± 14.99 (14–69)	39.78 ± 15.78 (14–69)
Sex (Male: Female)	1.38: 1	1:1
BMI	24.2 ± 3.47 (18–32)	24.28 ± 3.1 (16–31)
Comorbidities (0:1:2:>2)	56%: 22%: 16%: 6%	57%: 28%: 9%: 6%
Stone site (Right: Left)	3:2	1.6:1
Mean Stone size ± SD (Range) (mm)	23.88 ± 9.99 (12–62)	20.5 ± 6.92 (12–45)
Mean stone volume ± SD (Range) (cm [3]	16.16 ± 23.31 (1.29–114.91)	8.29 ± 10.28 (1.28–51.78)
Stone location	Total stone location: 64	
Pelvis/ureter	17 (26.6%)	17 (53.1%)
Lower pole	9 (14.1%)	1 (3.1%)
Inter polar	4 (6.3%)	0
Upper pole	22 (34.4%)	14 (34.4%)
Staghorn	12 (18.6%)	4 (12.5%)
Mean HU ± SD (Range)	1093 ± 232.83 (297–1504)	1045 ± 238.06 (297–1504)
Renal anomalies		
A: Nil	42 (84%)	26 (81%)
B: Horseshoe kidney	3 (6%)	1 (3.1%)
C: Malrotated kidney	1 (2%)	1 (3.1%)
D: Ectopic kidney	1 (2%)	1 (3.1%)
E: Diverticulum stone	3 (6%)	3 (9.4%)
Previous surgery		
Nil, PCNL, RIRS, Open Surgery	41 (82%), 4 (8%), 0, 5 (10%)	28(87.5%), 1(3.1%), 0, 3(9.4%)

Mean stone size was 23.88 ± 9.99 (range: 12–62) and mean stone volume was 16.16 ± 23.31 cm^3^ (range:1.29–114.91). Most common stone location was upper pole (*N* = 22, 34.4%) followed by pelvis/ureter (*N* = 17, 26.6%). Mean HU was 1093 ± 232.83 (range: 297–1504). Renal anomalies were present in 16% (*N* = 8) of patients which included three horseshoe kidney, three upper pole diverticulum, one malrotated kidney, and one ectopic kidney. Nine (18%) patients had history of previous surgery (PCNL:4 and open surgery: 5).

The procedure related details, outcomes, and complications are presented in Table 2. Spinal anesthesia was used for 98% (*N* = 49) cases, but as it was not effective in one case, it was converted to general anaesthesia. A total of 76 tracts were made for these 50 sPCNL cases. About 32 (64%), 11 (22%), 6 12%), and 1 (2%) patient had 1, 2, 3, and 4 tracts, respectively. Of the 26 additional tracts, there were 13 tracts in the mid and lower pole respectively.

**Table 2.** Procedural details, outcomes and complications (PCN – percutaneous nephrolithotomy, DJ – double J).

Parameter	Results (*n* = 50)	Only upper pole access (*n* = 32)
Anaesthesia	SA: 49, GA: 1	SA: 31, GA: 1
No of tracts	1: 32 (64%), 2: 11(22%), 3: 6(12%), 4: 1(2%)	Only upper pole access/tract
Tract location	Total tracts (76)	
Upper, Mid, Lower	50, 12, 13	
Relation to ribs (Supracoastal punctures)		
Sub coastal, Supracoastal, Supra 11^th^	15 (30%), 24 (48%), 11 (22%)	11 (34.4%), 16 (50%), 5 (15.6%)
Tract size	Total tracts (76)	
24 F, 22F, 20F, 16.5F	5 (6.6%), 21 (27.6%), 1 (1.3%),49 (64.5%)	0, 7 (22%), 0, 25(78%)
Lithotripsy modality		
Laser, Pneumatic, Combined	35 (70%), 4 (8%), 11 (22%)	29 (90.6%), 3 (9.4%), 0
Exit strategy	Tubeless (no PCN, DJ stent in all)	Tubeless (no PCN, DJ stent in all)
Duration of surgery (minutes)	67.92 ± 34.62 (20–150)	51.88 ± 25.68 (20–110)
Stone clearance	98.82%	99%
Flexible ureteroscope use (ECRIS)	6 (12%)	2 (6.25%)
Irrigation fluid (litres)	16.16 ± 8.31 (range: 3–39)	12.81 ± 6.74 (3–30)
Hemoglobin drop (gm/dl)	0.75 ± 0.42 (range: 0.2–2.3)	0.6 ± 0.33 (0.2–1.1)
Transfusion rate	2(4%)	0
Other organ injury	1 (2%), pleura	1 (3.1%), pleura
Complications (clavien-dindo)	I/II- 10 (20%), III- 1 (2%), Nil- 39 (78%)	1:- 5 (15.6%), 3:- 1 (3.1%%), nil:- 26 (81.3%)
Hospital stay (days)	2.14 ± 0.15 (range: 1–4)	2.06 ± 0.5 (2–4)
Readmissions	Nil	Nil

Supracoastal access was done in 70% (*N* = 35) cases, which included 11(22%) supra 11th rib punctures. Tract size larger than 20 F was made in 26 sPCNL access (34%), Maximum tract size was 24F. Laser was used as lithotripsy modality in 70% (*N* = 35) cases and pneumatic lithotripsy alone was used in only four (8%) cases. Exit strategy was tubeless in all the cases.

The mean duration of surgery was 67.92 ± 34.62 minutes (range: 20–150). Complete clearance was achieved in 98% cases. Flexible ureteroscope assistance (ECRIS) was required in six (12%) cases (antegrade in two patients and retrograde in four patients). Mean volume of normal saline used during the procedure was 16.16 ± 8.31 (range: 3–39) litres. The mean hemoglobin drop (preoperative and 24 hours postoperative) was 0.75 ± 0.42 (range:0.2–2.3) gm/dl. The complication rates for both supra and subcostal access were similar.

In our series, two (4%) patients required blood transfusion (one unit); one (2%) patient had pleura injury and effusion which resolved with USG guided aspiration. Ten (20%) of the patients had minor complications (Calvin-Dindo scale I/II), three (6%) had pain requiring analgesic, three (6%) had hematuria extending the hospital stay of which two needed blood transfusion), three, (6%) had fever on 1^st^ postoperative day needing oral antibiotics, and one patient had a 4 cm subcapsular hematoma which resolved spontaneously. The mean hospital stay was 2.14 ± 0.15 (range: 1–4) days and there were no readmissions.

## Discussion

To our knowledge, this is the first study evaluating the role of tubeless supine PCNL using an upper pole puncture. Upper pole is access of choice in prone PCNL at many centres around the world and the difficulty to access it in supine position has been cited as one of the drawbacks of sPCNL. In a small survey performed on supine access in complex situations and upper pole access, 45% of the urologists preferred a shift to prone in complex situations and additional 17% avoided sPCNL which required upper pole puncture.

PCNL is a time-tested gold standard procedure for renal stones larger than 2 cm, and it remains a viable alternative even for smaller stones [8]. Prone PCNL was the standard of care for treating renal stones and supine position had only limited indications. In prone position there are difficulties in ventilation and circulation, especially in morbidity obese patients. Furthermore, placing patients with spinal deformities (kyphoscoliosis), neck and limb contractures in prone position is difficult and risky. Patient repositioning requires more staff in the operating room and there is risk of displacement of urethral catheters and endotracheal tubes. Airway access to the anesthesiologist is limited in cases where conversation from spinal to general anesthesia is required. Surgeon and assistants are exposed to more radiation; it takes longer duration to complete the procedure and retrograde access to PCS is limited [9–11].

Supine PCNL has evolved significantly in recent years and is being performed with ease in centres around the world. It offers several advantages to anesthesiologist, better cardiovascular status and ventilation and accessibility to the airway at all times. Less manpower is needed during positioning, and simultaneous ureteroscopy and laparoscopy can also be performed. Surgeon is more comfortable (sitting) with c-arm and lithotripsy foot pedals in his control and less radiation exposure to the hands. The procedure itself takes less time both in positioning and stone clearance (spontaneous removal of fragments and less exchange of instruments) [11–14].

There were initial concerns about increased colon injury during sPCNL due to more lateral punctures, but Liu et al. [11] have reported lesser risk of colon injury (1 in 389 patients and reterorenal colon (2% vs 10%) in their meta-analysis establishing the safety of this technique. Small surgical field, collapse of collecting system, difficulty in intrarenal navigation, especially to the upper pole have been talking points against sPCNL, but after getting accustomed to the technique, these factors are mitigated [13]. In our experience with sPCNL, we did not find any difficulty in reaching the upper pole from the lower and even middle pole access.

The stone migration to upper pole during lithotripsy and need/fear of upper pole access have discouraged many urologists for sPCNL [15]. These stones can easily be retrieved from the lower and mid pole. The access to stones migrated to upper lateral calyx is a concern, but a maneuvre tilting the trunk to opposite side helps, as the stone falls to the medial upper calyx and can be retrieved. We needed upper calyceal puncture only in one out of 10 cases in our large series.

Considering the anatomy of ribs, pleura and lungs we have discovered that many upper calyceal punctures which are supracostal in prone position will be supra 11th in supine position, because ribs move downwards as we move anteriorly on the trunk. We target the upper lateral calyx for access rather than the upper medial calyx, as this leads to more medial punctures and more pleural injury. Sharma et el [16] reported 4.2% risk of pleural injury in their supracoastal pPCNLs, and Munver et al. [17] reported 16% overall pleural injury rate with supracostal access and 4.5% with an infracostal approach. Our study had only 2% rate of pleural injury in the supracostal access. Only one study by et el. [18] has 28 cases of supracostal puncture in supine PCNL without specifying upper pole access.

The mean age (39.8 years) and male predominance (1.38:1) in our study was similar to various other large series [18]. The mean stone size in our study (23.88 mm) was similar to Mulay et al. [19] (25.15 mm). 24% of cases were staghorn stones in our series compared to 31% in meta-analysis by Wu et al. [20]. 36% of the patients required more than one tract in our study emphasizing the safety of sPCNL in complex stones, Mulay et al. [19] reported multitract sPCNL in 12% of cases.

Supracoastal access was made in 48% and supra 11th access in another 22% of patients in our study, but no attempt was made to puncture the kidney in full inspiration as described by Goyal et al. [21] and others [14–16]. We believe that full inspiration opens up the pleural spaces and pleura moves downwards increasing its risk for injury. There was only a single case with reported pleural injury in our series.

Stone clearance was 98.82% in our series, and only two cases had residual fragment more than 4 mm at 1 month (USG and X-ray) which cleared without any ancillary procedure at 3 months follow up. Higher stone clearance when compared to previous studies [21,22] might be due to availability of flexible ureteroscope to aid in few situations where endoscopic combined intrarenal surgery (ECIRS) was done, and we used it in 6(12%) of cases.

Exit strategy was tubeless with Double J (DJ) stent in all patients, with a mean hemoglobin drop of 0.75 gm/dl and 2(4%) patients needing blood transfusion in our study which is better than 3–10% rate of acute bleeding requiring transfusion reported by Rosette et al. [3]. We used smaller tracts in our cases, mini PCNL in 50% and largest tract size was 24F which might amount to lesser bleeding and safe tubeless exit.

Reduced operative time is a well-known advantage of sPCNL. Our mean operative time was 67.92 minutes (range: 20–150 minutes). Mulay et al. [19] reported time from initial position to completion of 72.24 minutes in sPCNL compared to 88.12 minutes in prone position, and the time from puncture to finish was also shorter in sPCNL (51 vs 56 minutes). Spontaneous gravity dependent removal of fragments and less need to use graspers makes the sPCNL faster. Mean Hospital stay was 2.14 days in our study, similar to other reported studies [19,20].

The accessibility of both anterograde and retrograde access to PCS makes the sPCNL more attractive [23]. In the era of Flexible ureteroscopy and ECRIS, limiting the sPCNL to simple cases is not needed. In cases where ureteric retrograde access could not be achieved, or in long, narrow upper pole diverticular stone, the technique of upper pole access will yield good and safe results.

This study has limitations as a retrospective series, in a single-centre, with a small diverse group of patients. Post-procedural CT scan would also have been more definitive to assess SFR. There is a lack of a pPCNL comparison arm and ideally future studies should look at a randomized trial to compare the positioning as well as tract site and size [24,25]. Perhaps a role of using nomograms and measuring patient quality of life should also be built into these studies [26,27].

## Conclusion

Supine PCNL is a feasible and safe approach for upper pole access and has the advantage of performing simultaneous flexible ureteroscopy. It can be performed with the same ease as in prone position and has excellent outcomes in terms of stone clearance with small risk of mostly minor complications. Furthermore, it can be done under a spinal anesthesia with a tubeless exit, offering a safe strategy in complex patients.
